# Clinical Conundrum of Acute Hepatitis B With Concurrent Hepatitis E Infection Leading to Severe Acute Liver Injury

**DOI:** 10.7759/cureus.35216

**Published:** 2023-02-20

**Authors:** Faryal Altaf, Zaheer A Qureshi, Sameer Kandhi, Misbahuddin Khaja

**Affiliations:** 1 Internal Medicine, Icahn School of Medicine at Mount Sinai/BronxCare Health System, New York, USA; 2 Internal Medicine, The Frank H. Netter M.D. School of Medicine at Quinnipiac University, North Haven, USA; 3 Internal Medicine, BronxCare Health System, New York, USA; 4 Internal Medicine/Pulmonary Critical Care, Icahn School of Medicine at Mount Sinai/BronxCare Health System, New York, USA

**Keywords:** hepatitis b, hepatitis e, acute liver failure, cirrhosis, liver and gall bladder disease, acute liver failure (alf), hepatitis e virus, viral hepatitis b

## Abstract

Acute liver injury in the setting of acute fulminant hepatitis caused by the hepatitis B virus (HBV) can occur both during primary infection and after chronic HBV reactivation. Guidelines recommend considering antiviral therapy in both cases. Antiviral therapy with a nucleoside analog may be beneficial in patients with acute liver failure from acute HBV infection, though not all studies have shown a benefit. This is a case of a 53-year-old woman with a past medical history of untreated hepatitis C with undetectable viral load and right breast cancer status post lumpectomy, who presented to the emergency department with complaints of yellowish skin and sclera discoloration with right upper quadrant pain for one week. She was a known intravenous drug abuser and binge alcohol user. Her labs were positive for hepatitis B, hepatitis E, and hepatitis C viruses. She also had elevated liver enzymes with hyperbilirubinemia showing severe acute liver injury. Computed tomography of the abdomen and pelvis with contrast was normal, and the abdominal ultrasound showed homogenous echotexture of the liver without a focal lesion. The patient was diagnosed with acute fulminant hepatitis B. After initial hemodynamic stabilization, N-acetylcysteine (NAC) and tenofovir were started, and transaminases were followed. Liver function tests showed a downtrend, and, in a few weeks, they came to baseline. Hepatitis B viral load became undetectable as well. Acute hepatitis B infection is seldom treated. The presented case depicts the use of tenofovir in the setting of severe acute liver injury due to hepatitis B. Starting antiviral therapy (especially tenofovir disoproxil fumarate) early in the disease course was shown to have very assuring results with complete resolution of symptoms and normalization of liver function tests. The treatment protocol for acute HBV deserves further investigation.

## Introduction

Hepatitis B virus (HBV) infection is the 10th leading cause of death worldwide [1]. When an individual is exposed to HBV, it leads to a vigorous and broad immune response leading to self-limited infection, resulting in acute hepatitis. An aggressive response can lead to fulminant hepatitis [1]. Acute hepatitis B in approximately 90-95% of cases recovers spontaneously, but in about 1% of cases, it can manifest as fulminant hepatitis, which is associated with a high fatality rate and largely relies on liver transplantation as a life-saving intervention [1]. Antiviral drugs are not routinely recommended for the management of acute hepatitis B. However, treatment with such drugs is the mainstay of management of cases of severe acute or reactivated hepatitis and should be started as a matter of urgency treatment to prevent death. We demonstrate a case of a woman who had chronic hepatitis C virus (HCV) with undetectable HCV RNA and became infected with viral hepatitis E and hepatitis B. She was started on tenofovir due to acute fulminant liver failure due to hepatitis B leading to down-trending transaminases and decreasing viral load.

## Case presentation

A 53-year-old woman presented to the emergency department with one week of yellowish skin, scleral discoloration, and right upper quadrant (RUQ) pain. She described the pain as dull, non-radiating, constant, and 5/10 in intensity, without aggregating factors. The pain was associated with high-grade fever with chills. She denied nausea, vomiting, diarrhea, constipation, loss of appetite, early satiety, unintentional weight loss, hematemesis, melena, hematochezia, use of nonsteroidal anti-inflammatory drugs, acetaminophen, and herbal medications, dizziness, or lightheadedness. Her comorbid conditions included untreated hepatitis C, and right breast cancer status post lumpectomy. Her social history was significant for the use of intravenous drug injections, smoking, and binge alcohol drinking. She did not remember the time of her last drink. Travel history was negative for recent travel. There was no significant history of malignancy or liver disease. Currently, she has been living in an alcohol and drug rehabilitation facility for the last two months.

**Figure 1:**
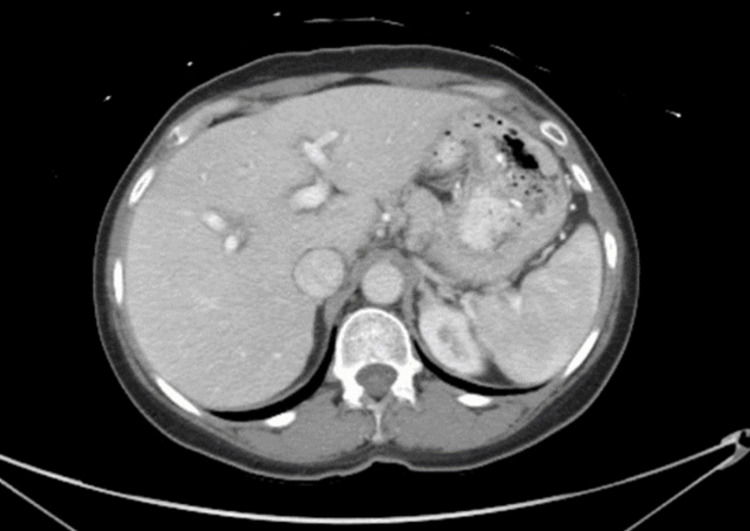
CT of the abdomen and pelvis with contrast showing a normal liver architecture

**Figure 2:**
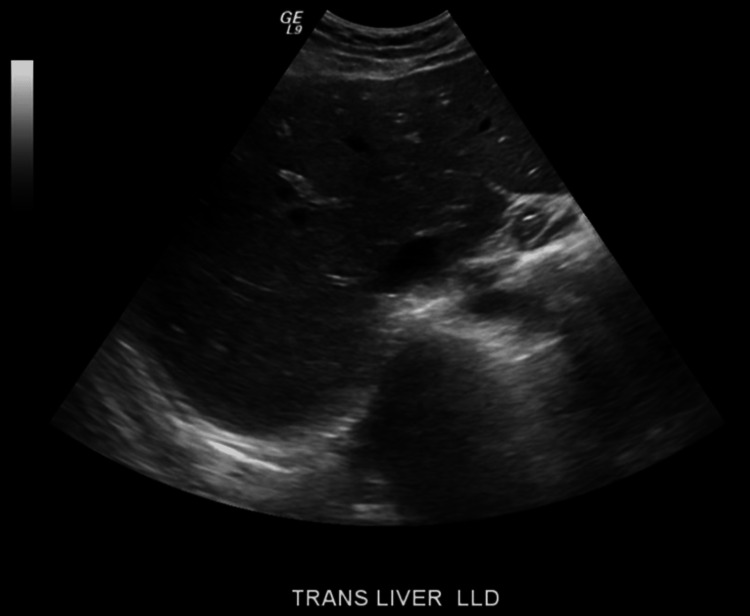
Ultrasound of the liver showing a normal liver architecture

On admission, she was hemodynamically stable with mild hypoglycemia. On physical examination, she had scleral icterus on the eye exam and mild RUQ tenderness in the abdominal exam. The rest of the physical examination was unremarkable. Laboratory findings are shown in Tables 1, 2. Labs on admission showed elevated liver enzymes, increased partial thromboplastin time, elevated international normalized ratio (INR), and increased ferritin level. She had aspartate aminotransferase (AST) of 2484 U/L, alanine transaminase (ALT) of 1608 U/L, alkaline phosphatase (ALP) of 235 U/L, total bilirubin of 6.6 mg/dL, direct bilirubin of 5.3 mg/dL, partial thromboplastin time of 39.8 seconds, INR of 1.2, serum ammonia of 55, and ferritin of 1655. No previous liver function tests were available to compare baseline values. Computed tomography (CT) of the abdomen and pelvis with contrast showed no significant findings (Figure 1). Abdominal ultrasound was done showing homogenous echotexture liver without focal lesion with normal common bile duct (CBD) diameter and no obvious cholelithiasis or choledocholithiasis (Figure 2). Chest X-ray was normal as well.

**Table 1:** Laboratory data with the trend

Lab results (reference range)	Reference range	Day 1	Day 3	Day 7
Hemoglobin, g/dL	12-15	12.1	12.1	11
White cell count, k/UL	4-10	6.5	5.8	6.2
Neutrophil %	40-60	58.6	58.8	68.5
Lymphocyte %	20-40	27.5	24.9	24.2
Platelets, k/UL	150-450	297	280	252
Sodium, mEq/L	135-145	136	137	137
Potassium, mEq/L	3.5-5	4	4.4	4.4
Bicarbonate, mEq/L	22-28	22	23	23
Blood urea nitrogen (BUN), mg/dL	6-24	7	11	8
Creatinine, mg/dL	0.5-1.0	0.4	0.3	0.4
Calcium, mmol/L	8.5-10	8.2	9	8.8
Total protein, g/dL	6-8	6.1	6.8	6.5
Albumin, g/dL	3.5-5.5	3	3.3	3.2
Aspartate aminotransferase (AST), U/L	8-33	2484	2894	1110
Alanine transaminase (ALT), U/L	4-36	1608	2051	406
Alkaline phosphatase (ALP), U/L	45-145	235	235	208
Total bilirubin/direct bilirubin, mg/dL	0.1-1.2/<0.3	6.6/5.3	8.2/6.1	2.6/1.6
Prothrombin time, second	11-13.5	13	13.2	11.1
Partial thromboplastin time, second	25-35	39.8	62.9	41.6
International normalized ratio (INR)	<1.1	1.2	1.2	1
Urine analysis		Normal		
Lactic acid	<2	1.7		

**Table 2:** Miscellaneous laboratory data

Lab results	Reference range	
Antinuclear antibodies (ANA)		Negative
Ceruloplasmin, serum	14-40	27
Ferritin, serum	11-307	1655
Gamma-glutamyl transferase (GGT), U/L	5-40	171
Anti-smooth muscle antibody (ASMA)		Positive
Human Immunodeficiency virus (HIV)		Negative
Urinary toxicology screen		Negative
Urinary analysis		Negative
Ammonia, serum, µ/dL	15-45	55
Anti-mitochondrial antibody (AMA)		Negative
Acetaminophen level, mcg/mL		<14.9
Acetylsalicylic acid level		<3.1
Anti-liver kidney microsomal antibody		Negative
Ethanol, mg/dL	<10	<10

The gastroenterology (GI) team was consulted for assistance with further workup and management of severe acute liver injury. The GI team recommended a complete baseline workup, including a complete hepatic panel and viral hepatitis serology. Her hepatic panel showed that she was immune to hepatitis A. She was positive for hepatitis B, hepatitis C, hepatitis E, and anti-smooth muscle antibodies (Table 3). Furthermore, she was negative for hepatitis D, HIV, acetaminophen toxicity, and aspirin toxicity. The rest of the hepatic panel is shown in Table 3. A further detailed panel showed a hepatitis B viral load of 910,731 and a hepatitis C viral load was undetectable.

**Table 3:** Viral hepatitis panel HAV: hepatitis A virus; EIA: enzyme immunoassay; PCR: polymerase chain reaction.

Hepatitis A total antibody	Detected
Hepatitis A (anti-HAV) IgM	Not detected
Hepatitis B core total antibody	Detected
Hepatitis B core IgM antigen	Detected
Hepatitis B surface antigen	Detected
Hepatitis B surface antibody	Not detected
Hepatitis Be antibody	Not detected
Hepatitis Be antigen	Detected
Hepatitis B viral DNA Cop/mL	5.96
Hepatitis B viral DNA IU/ML	910,731
Hepatitis B viral DNA	Detected
Hepatitis B surface antibody, QL.	Not detected
HCV antibody-EIA	Detected
Hepatitis C (HCV) RNA, IU/ML (Quan)	Not detected
HCV RNA by PCR (Quan)	Not detected
Hepatitis E antibody, (IgG)	Not detected
Hepatitis E antibody, (IgM)	Detected
Hepatitis D antibody, total	Not detected

Abnormal liver function tests (LFTs) were suspected due to acute infection by hepatitis B leading to severe acute liver injury. Though the patient had no alteration in sensorium, she was deemed to be at increased risk of impending acute liver failure (ALF), which was evident from her worsening INR and serum albumin levels. After discussion with the liver transplant center on the second day of admission, the GI team decided to start her on N-acetylcysteine (NAC) treatment as per protocol, i.e., NAC 150 mg/kg over 60 minutes, followed by 50 mg/kg over four hours (second dose) and 100 mg/kg over 16 hours (third dose) in combination with tenofovir 300 mg, with recommendations to immediately transfer to the liver transplant center in case of worsening sensorium or liver dysfunction. NAC serves as a prodrug to L-cysteine, which is a precursor to the biological antioxidant glutathione. Hence, the administration of acetylcysteine replenishes glutathione stores. NAC is used in ALF, as well as some lung and kidney diseases, mostly off-label. Tenofovir disoproxil is a nucleotide analog reverse-transcriptase inhibitor (NRTI) used to treat hepatitis B and HIV. She was also started on intravenous fluids, thiamine, folic acid, and multivitamins as well. Liver function tests and coagulation profiles were trended very closely.

**Figure 3:**
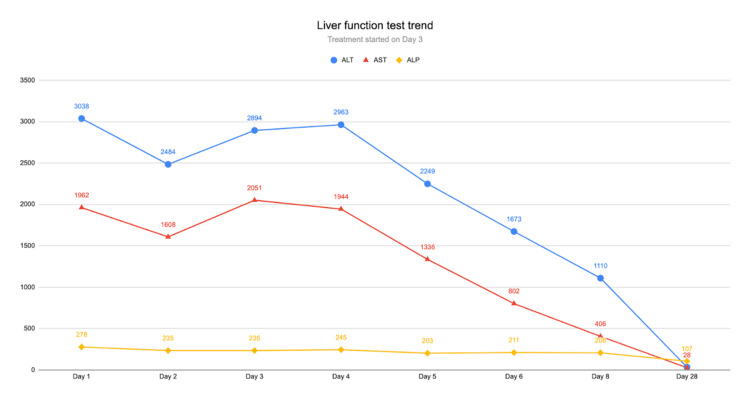
Graphical representation of the patient's liver function test in U/L (y-axis) against time in days (x-axis) ALT: alanine transaminase; AST: aspartate aminotransferase; ALP: alkaline phosphatase.

On the fourth day of admission, LFTs started to downtrend. She was feeling better as well. On day eight, she was discharged on tenofovir 300 mg once a day with an outpatient GI clinic appointment after three weeks. At the clinic visit, her LFTs showed a marked improvement, and LFTs were at baseline within two months. Her hepatitis b viral load down trended from 910,731 to <20 (undetectable). Her last LFTs were AST of 35 U/L, ALT of 28 U/L, and ALP of 107U/L. The trend of LFTs from day one of hospitalization to day eight is shown in Figure 3. During the treatment course, no features of tenofovir-associated side effects were observed in the patient. She tolerated the treatment well.

## Discussion

ALF is a rare and challenging clinical syndrome. The course is variable, and the mortality rate is very high. As per the American Association for the Study of Liver Diseases (AASLD), ALF is characterized by evidence of coagulopathy (INR ≥ 1.5) and the presence of an altered sensorium (encephalopathy) without pre-existing cirrhosis and with a duration of symptoms of less than 26 weeks [2]. Acute liver injury, on the other hand, is characterized by worsening elevated liver enzymes with hyperbilirubinemia and coagulopathy with no presence of encephalopathy. Acute liver injury/ALF can result from various causes, especially overdosing with acetaminophen, viral hepatitis, alcoholic hepatitis, autoimmune hepatitis, and other causes. Viral hepatitis is still one of the key causes of ALF. Hepatitis B was regarded as the most common cause of ALF [3]. The use of vaccination against HBV leads to a decrease in incidence. There are still some patients who present with HBV-related acute fulminant liver failure [4]. In adults, HBV is usually asymptomatic. According to the WHO, ¼ of the world's population is estimated to have experienced HBV infection, and 3% have chronic HBV [5]. Hepatitis E virus (HEV) could cause superinfection in HBV patients as well. The outcome of HEV superinfection in HBV patients appears more severe than the standalone infection [6].

In areas with high hepatitis B prevalence, it spreads from mother to child at birth. In developed countries, it is mainly spread by sexual transmission [7]. Other modes include the reuse of needles and syringes, tattooing, use of razors, and similar objects that are contaminated with infected blood [8]. The diagnosis of acute hepatitis B infection is based on the detection of hepatitis B surface antigen and IgM antibody to hepatitis B core antigen. As a rule, those patients are treated who have developed a coagulopathy (INR > 1.5) and those with persistent symptoms or marked jaundice (bilirubin > 3 mg/dL) for more than four weeks after presentation [9]. Other clinical manifestations may include jaundice, hepatomegaly, and RUQ tenderness [10]. As the liver injury progresses, anicteric patients may develop jaundice, and those with mild mental status changes may become confused or even comatose precipitating ALF. Patients with ALF may have abnormal laboratory tests, including prolonged prothrombin time (PT)/INR, elevated AST/ALT levels, elevated bilirubin levels, or low platelet count [11]. These patients may have acute kidney injury (AKI), which could further complicate ALF. The preferred imaging modalities for diagnosis of fulminant hepatic injury are CT and ultrasound with Doppler imaging of the abdomen. Finally, if laboratory tests and imaging are non-conclusive, a liver biopsy could be done.

Currently, in the United States, spontaneous survival for ALF is approximately 45%, liver transplantation is 25%, and death without transplantation is 30% in adults [12]. The general management of the patient with ALF includes proper setting, monitoring for worsening liver function tests, treating complications, and nutritional support. The mainstay for ALF treatment of the liver injury caused by underlying HBV infection, with proven benefits, is NAC, antiviral therapy, and liver transplant [13]. There is very little evidence supporting antiviral therapy in the early stages of treatment of acute fulminant hepatitis B-related liver failure. The evidence mainly includes expired patients or patients who required transplantation despite therapy with nucleoside analogs (NA) [14]. Jochum et al. [4] showed that immediate treatment of HBV-induced ALF with entecavir, a nucleoside analog very similar to tenofovir, is well tolerated and beneficially affects the course of the disease. Tenofovir or entecavir are acceptable options given as monotherapy and can be stopped after confirmation that the patient has cleared hepatitis B surface antigen (HBsAg) [15]. However, to date, there is no evidence that suggests NA is the best therapy in this setting, as randomized control trial comparing different NAs is lacking.

Tenofovir disoproxil fumarate (TDF) is an oral prodrug of tenofovir approved for chronic hepatitis B treatment by the FDA since 2008 [16]. TDF has been shown to be highly effective in suppressing HBV replication in both LAM (lamivudine)-naïve and LAM-resistant patients [17]. According to the results of a prospective randomized controlled trial comparing the use of TDF with a placebo for the treatment of HBV-related acute‐on‐chronic liver injury, the use of tenofovir early in the disease course was able to significantly reduce HBV DNA levels and decrease both Child-Turcotte-Pugh (CTP) and Model for End-Stage Liver Disease (MELD) scores, thus improving survival in those patients [18].

The decision to proceed with liver transplantation depends on the probability of spontaneous hepatic recovery. The goal should be to differentiate patients who are likely to benefit from liver transplantation from those likely to recover spontaneously. The King's College criteria are the most widely used for selecting a patient for a liver transplant, but also Clichy's criteria, and more recently, the MELD score, have all been used [19]. It includes a PT greater than 100 seconds. Otherwise, age less than 10 or greater than 40 years, unfavorable disease etiology, such as non-A, non-B viral hepatitis, idiosyncratic drug reactions, Wilson disease or duration of jaundice before the development of encephalopathy < seven days or PT greater than 50 seconds or serum bilirubin greater than 18 mg/dL. The MELD score has also been used to predict survival among patients with ALF. Overall, the survival rate in patients treated for ALF is <60% [20]. Approximately 55% will survive without the need for a liver transplant. Without liver transplantation, approximately one year of survival is expected [21].

Clinicians often encounter patients with HBV-related acute fulminant hepatic injury with an increased risk of precipitation to acute hepatic failure with a crucial decision about the treatment course. Do we have a window of treatment trial with NA, or patient should be referred for a liver transplant immediately? Malignant infiltration of the liver should be considered in the differential diagnosis of severe acute liver injury [22-26].

This case brings forth an exciting patient with HBV-related acute fulminant hepatic injury, treated with TDF 300 mg once a day, resulting in complete resolution of symptoms in two months. The AASLD also recommends considering nucleos(t)ide analogs as a possible treatment option for hepatitis B ALF [4]. There is a need for randomized control trials for the need for NAC. In the absence of reliable, evidence-based data, hepatologists could treat their patients with severe acute hepatitis B with lamivudine or the most potent antivirals entecavir or tenofovir. Concurrent infection with hepatitis E leading to ALF has never been studied.

## Conclusions

Acute hepatitis B infection is seldom treated. It can lead to acute liver failure. Starting antiviral therapy such as TDF early in the disease course is a very assuring treatment plan resulting in complete resolution of symptoms and normalization of liver function tests. The treatment protocol for acute HBV deserves further investigation. Hepatitis E superinfection on chronic hepatitis B can contribute to significant morbidity and mortality, especially in patients with pre-existing cirrhosis. Thus, in the absence of reliable, evidence-based data, hepatologists could treat their patients with severe acute hepatitis B with lamivudine or the most potent antivirals entecavir or tenofovir.
